# The West Ham Hospital's Jubilee

**Published:** 1912-05-25

**Authors:** 


### The West Ham Hospital's Jubilee.

At a meeting held in the Town Hall, West Ham, last Tuesday, the Mayor of West Ham appealed for suggestions as to how best to make the receipts of the hospital a record for this the jubilee year. He stated that he remembered the old infirmary with its green baize covered door in the Romford Road many years back, and that the progress of the hospital had been very great—whether adequately great for the needs of the enormous population it was not for him to say.

Mr. T. A. Cook, the chairman of the hospital, then said he had no particular suggestions, but recalled the first meeting of the Committee, which consisted of one baronet (Sir T. F. Buxton), three M.P.s, five J.P.s, one captain R.N. (Richard Pelly), and one major (Silver), and that the objects of the West Ham, Stratford, and South Essex Dispensary were "to alleviate the sufferings of the poor when deprived not only of the comfort of health, but as a necessary consequence of the means of subsistence." In 1867 cholera visited the district. The institution was open day and night. The house surgeon heroically attended constantly, and the Board of Guardians recognising the value of the institution defrayed the expenses of a dispenser for the year. He reminded those present that in 1869 one guinea voluntarily and unexpectedly was contributed by the porters and men at Stratford Station. In 1871 Mr. Thomas Lloyd, of St. Mary's Hospital, Paddington, was appointed dispenser; and single-handed he had dealt with the increased work until the current year, when the Committee appointed an assistant. Since 1904, Mr. Cook proceeded, a great effort had been made to extend the hospital, and £40,000 has been raised to that end. The hospital now had a full complement of just over 100 beds and had recently accommodated 115 in-patients in one night.

Various suggestions were made, and the most important resolutions carried were :—(1) To hold a bazaar during the current year; (2) the Mayor promised to give a Jubilee Ball in aid of the hospital, from which he would relieve hospital workers of any detail work; (3) there was also a suggestion to raise a Million Pennies Fund in the borough and neighbourhood. With a population of 300,000 in the borough and close on a million souls in the neighbouring districts served by the hospital, it was urged that to raise such a fund should be easy.

